# Hand-Held Near-Infrared Spectroscopy for Authentication of Fengdous and Quantitative Analysis of Mulberry Fruits

**DOI:** 10.3389/fpls.2019.01548

**Published:** 2019-11-27

**Authors:** Hui Yan, Yi-Chao Xu, Heinz W. Siesler, Bang-Xing Han, Guo-Zheng Zhang

**Affiliations:** ^1^School of Biotechnology, Jiangsu University of Science and Technology, Zhenjiang, China; ^2^Department of Physical Chemistry, University of Duisburg-Essen, Essen, Germany; ^3^College of Biological and Pharmaceutical Engineering, West Anhui University, Lu’ an, China

**Keywords:** hand-held spectrometers, instrumentation, near-infrared (NIR), qualitative and quantitative analysis, authentication of fengdous, nutritional parameters of mulberry fruits

## Abstract

Recently, miniaturization of Raman, mid-infrared (MIR) and near-infrared (NIR) spectrometers have made substantial progress, and marketing companies predict this segment of instrumentation a significant growth rate within the next few years. This increase will be based on a more frequent implementation for industrial quality and process control and a broader adoption of spectrometers for in-the-field testing, on-site measurements, and every-day-life consumer applications. The reduction in size, however, must not lead to compromises in measurement performance and the hand-held instrumentation will only have a real impact if spectra of comparable quality to laboratory spectrometers can be obtained. The present communication will, on the one hand, explain the instrumental reasons why NIR spectroscopy is presently the most advanced technique regarding miniaturization and on the other hand, it will emphasize the impact of NIR spectroscopy for plant analysis by discussing in some detail a qualitative and a quantitative application example.

## Introduction

Miniaturization of vibrational spectrometers started more than two decades ago, but only within the last decade real hand-held Raman, MIR and NIR scanning spectrometers have become commercially available and have been utilized for a broad range of analytical applications (Sorak et al., 2012; Guillemain et al., 2017; Crocombe, 2018; Karunathilaka et al., 2018; Soriano-Disla et al., 2018; Vargas Jentzsch et al., 2018). While the weight of the majority of Raman and MIR spectrometers is still in the s1 kg range, the miniaturization of NIR spectrometers has advanced down to the ∼100 g level and developments are underway to integrate them into mobile phones (Tino et al., 2016). Furthermore, miniaturized NIR systems have recently reached the <1,000 US$ level. Therefore, only the acquisition of NIR systems can be taken into consideration for private use whereas hand-held Raman and MIR spectrometers will be restricted to industrial, military or homeland security applications and public use by first responders, customs or environmental institutions.

Because of the substantial progress in the miniaturization of near-infrared spectrometers in combination with a drastic cost reduction, marketing experts predict this type of instrumentation a significant growth rate. These trends have made hand-held NIR spectroscopy also attractive for everyday life consumer applications of a new, non-expert user community ranging from food testing to the detection of fraud and adulteration in a broad area of materials. Notwithstanding this wide-spread application range of hand-held NIR spectroscopy, the focus of this communication will be for plant analytical aspects only. The discussion of a qualitative and a quantitative analytical problem shall serve as examples, to demonstrate the vital role that hand-held NIR spectroscopy will play in the near future for plant analysis.

Before these selected qualitative and quantitative case studies are discussed, however, an overview of the various instrumental features of the most frequently used hand-held NIR spectrometers will be given.

## Instrumentation

The recent progress in miniaturization of hand-held NIR spectrometers has taken advantage of new micro-technologies such as MEMS (Micro-Electro-Mechanical Systems), MOEMS (Micro-Opto-Electro- Mechanical Systems), DMD^™^ (digital mirror device), or LVFs (Linear Variable Filters) and has led to a drastic reduction of spectrometer size (the weight of the spectrometers discussed in this communication varies between 100 and 200 g) while allowing excellent performance due to the high-precision implementation of essential elements in the final device (Wolffenbuttel, 2005). High-volume manufacturability will further reduce costs and thereby contribute towards broader dissemination of such instruments. In what follows the specific instrumental features of four different hand-held NIR spectrometers will be shortly outlined.

Based on the type of detector, the hand-held NIR spectrometers can be classified in the two categories of array-detector and single-detector instruments (Wolffenbuttel, 2005). Probably the first commercial, real hand-held NIR spectrometer (VIAVI MicroNIR 1700 (formerly JDSU), Santa Rosa, CA, USA) has an array detector that covers the wavelength range from 908 to 1,676 nm and uses an LVF as a monochromator. It has so far been used for a multiplicity of applications ranging from authentication of seafood and determination of food nutrients to the analysis of hydrocarbon contaminants in soil and authentication and quantitative determination of pharmaceutical drugs (Altinpinar et al., 2013; O’Brien et al., 2013; Jantra et al., 2017; Yan and Siesler, 2018b). However, compared to an array detector, the price for a single detector is much lower, and in an attempt to further reduce the hardware costs, new developments focus on systems with single detectors. Thus, the DLP NIRscan Nano EVM (Dallas, TX, USA), for example, is based on Texas Instruments’ DMD^™^ in combination with a grating and a single-element detector and also covers the wavelength range from 900 to 1,701 nm. Very recently a MEMS-based FT-NIR instrument, that contains a single-chip Michelson interferometer with a monolithic opto-electro-mechanical structure has been introduced by Si-Ware Systems (Cairo, Egypt). Contrary to most of the other handheld spectrometers, this instrument can scan FT-NIR spectra over the extended range from 1,298 to 2,606 nm. Finally, Spectral Engines (Helsinki, Finland) developed miniaturized NIR spectrometers, that are based on a tunable Fabry-Perot interferometer. In order to cover the NIR wavelength region 1,350–2,450 nm, however, four spectrometers are required.

**Figure 1:**
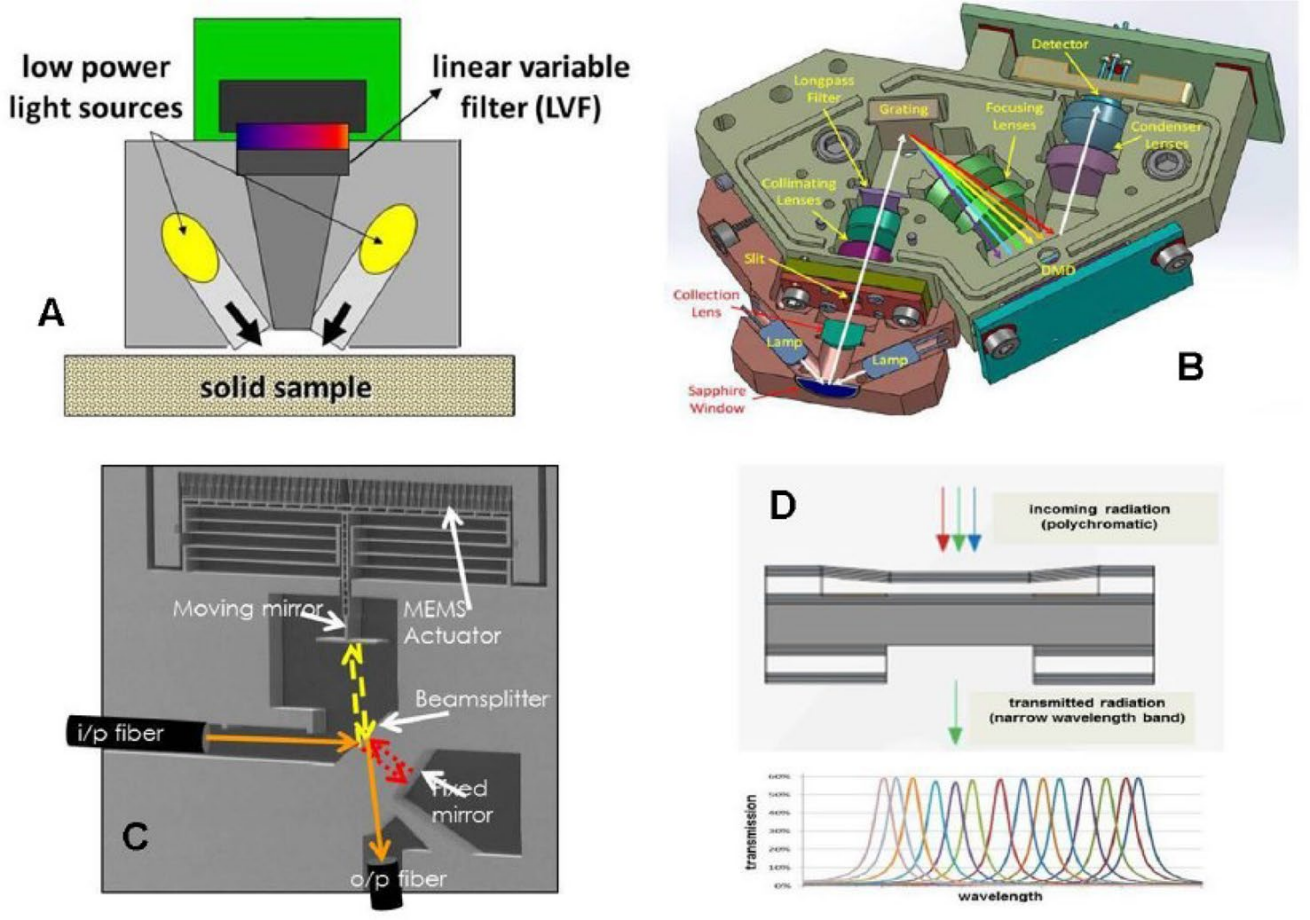
The optical schemes of hand-held NIR spectrometers based on different monochromator principles **(A)** VIAVI MicroNIR 1700, linear variable filter; **(B)** DLP NIRscan Nano EVM with Texas Instruments´ digital micromirror device (DMD™); **(C)** Si-Ware Systems, MEMS-based FT-NIR spectrometer; **(D)** Spectral Engines NIR spectrometer with tunable Fabry-Perot interferometer.

The schematic principles of the different monochromator designs of the described NIR spectrometers are summarized in **Figure 1**.

## Applications

Although the NIR technique is usually applied for a broad range of industrial material quality and control applications (Grassi et al., 2018; Piao et al., 2018; Silva et al., 2018; Yan and Siesler, 2018a; Yan and Siesler, 2018b), the present communication is targeted at practical, everyday life applications in order to attract the attention of a prospective non-expert user community. These days, qualitative and quantitative analysis is more than ever needed also by ordinary people. Because both fraud and adulteration are widely spread, and public health awareness has grown strongly over the last years, the control of nutritional parameters of everyday life food and pharmaceuticals has become an important issue. Therefore, the progress in miniaturization and increasing affordability of hand-held NIR spectrometers make them an attractive tool to fight the above evils efficiently in the public domain.

To demonstrate the potential of hand-held NIR spectrometers for plant analysis, a qualitative and a quantitative application example will be presented here.

### Identification of Fengdous

**Figure 2:**
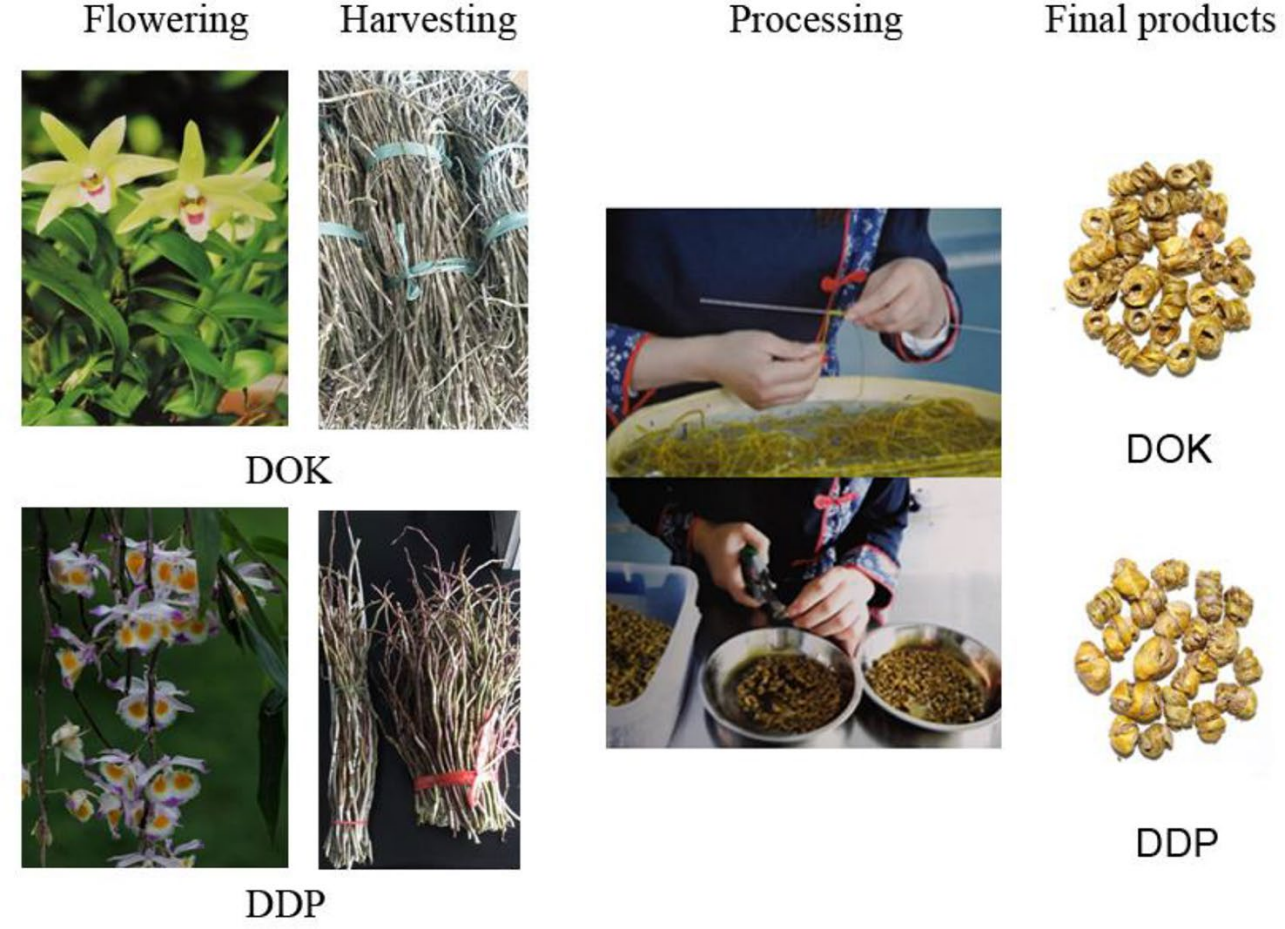
Schematic diagram of the fengdou processing.

In China, the stem of the Dendrobium is processed into a fengdou (**Figure 2**), that is considered a convenient dosage form of not only a valuable health-care food but also Chinese traditional medicine (TCM) with efficacy in liver protection, treatment of pharyngitis and many other diseases (Chen and Guo, 2001). Fengdou processed from *Dendrobium officinale Kimura et Migo* (DOK) have not only high medicinal value but are also in short supply, and, are very expensive. Therefore, it would be desirable to discriminate them from fengdou based on *Dendrobium devonianum Paxt* (DDP) with lower efficacy and correspondingly much lower (1/4–1/5) price. However, this is not possible by visual inspection only (**Figure 2**).

Because of the high public interest, an analytical method based on hand-held NIR spectroscopy with the DLP NIRscan Nano EVM system in combination with a partial least squares discriminant analysis (PLS-DA) evaluation method was developed, to rapidly discriminate fengdou processed either from DOK or DDP.

#### Materials and Methods

##### Samples

A total of 468 fengdou samples based on DOK (288) and DDP (180) were collected from Luosiwan (Yunnan, China), and the calibration and validation sets were randomly distributed at a ratio of 2:1.

##### Measurement of Spectra

**Figure 3:**
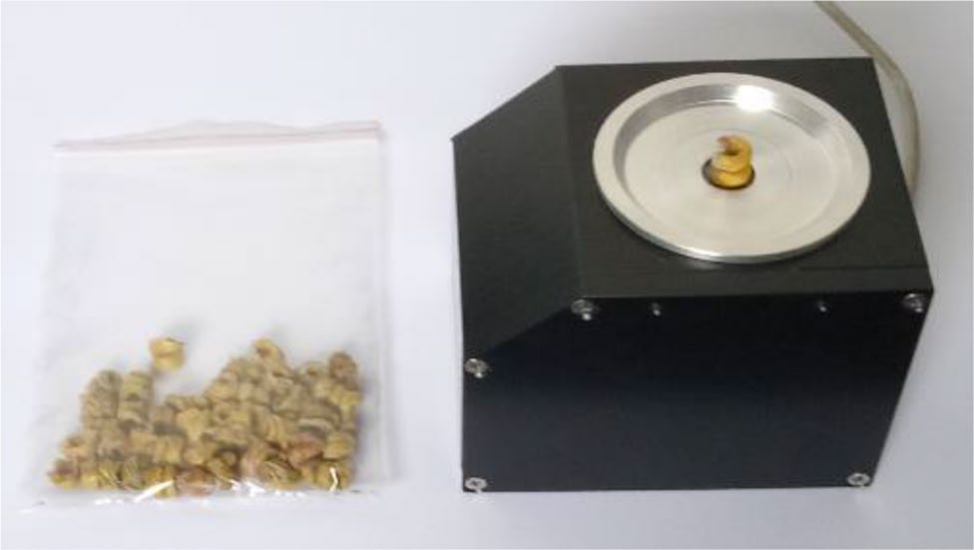
The sample presentation to record NIR spectra of fengdous.

NIR spectra were collected with the DLP NIRScan Nano EVM spectrometer by accumulating 32 scans in the wavelength range of 909–1,649 nm (209 wavelength variables) in approximately 7 s. After each measurement, the sample was rotated for approximately 120°, and the average of three spectra was then used as the final raw spectrum (**Figure 3**). A certified reflection standard (Labsphere, North Sutton, NA, USA) was used to measure the reference spectrum.

##### Evaluation of Spectra

###### Spectral Pretreatment

Due to the fact that NIR spectra frequently contain interferences of background information, drift, and noise, the raw NIR spectra were subjected to spectral preprocessing. For this purpose, the first derivative based on a Savitzky Golay smoothing procedure with a five data point window and a 2nd order polynomial followed by a standard normal variate (SNV) transformation as a scatter correction was used.

###### Competitive Adaptive Reweighted Sampling

In NIR spectroscopy, the spectral information is not evenly distributed over the whole wavelength range under investigation. Some data may be superimposed by noise or contain irrelevant information, that can decrease the performance of the calibration models. Therefore, the selection of the informative variables is a significant preprocessing step (Li et al., 2009; Li et al., 2013; Yun et al., 2013). In this work, the competitive adaptive reweighted sampling (CARS), based on the simple but effective principle “survival of the fittest” was applied to select the optimal combinations of spectral variables (Zhang et al., 2015). Compared to the moving window algorithm and Monte Carlo uninformative variable elimination procedure, CARS shows a strong capability of increasing the predictive accuracy (Li et al., 2009). For the present analysis, the CARS was run by the libPLS toolbox (www.libpls.net) based on the best combination of pretreated spectra.

##### PLSDA Analysis

The informative spectral variables determined by CARS were used to develop classification models with the PLS-DA. PLS-DA is a linear classification method that is based on the well-known partial least-squares (PLS) regression. In this work, the leave-one-out (LOO) method was applied to obtain the optimal number of latent variables (LVs) of each model, and the LVs with the lowest root mean square error (RMSE) of cross-validation set (RMSECV) were employed to establish the PLS-DA classification model.

The indices of class accuracy, which are described in the following equation, were calculated to evaluate the performance of each classification model. The higher the accuracy values, the better the predictive ability of the classification model:

$$Class\;accuracy=\frac{Number\;of\;correct\;assignments\;of\;each\;class}{Total\;sample\;number\;of\;each\;class\;tested}.$$

All calculations were performed in MATLAB environment (R2009, Mathworks, Natick, MA, USA) and PLS-DA models were built using the “PLS Toolbox 6.21” from Eigenvector Research (Manson, WA, USA).

#### Results and Discussion

##### NIR Spectra

**Figure 4:**
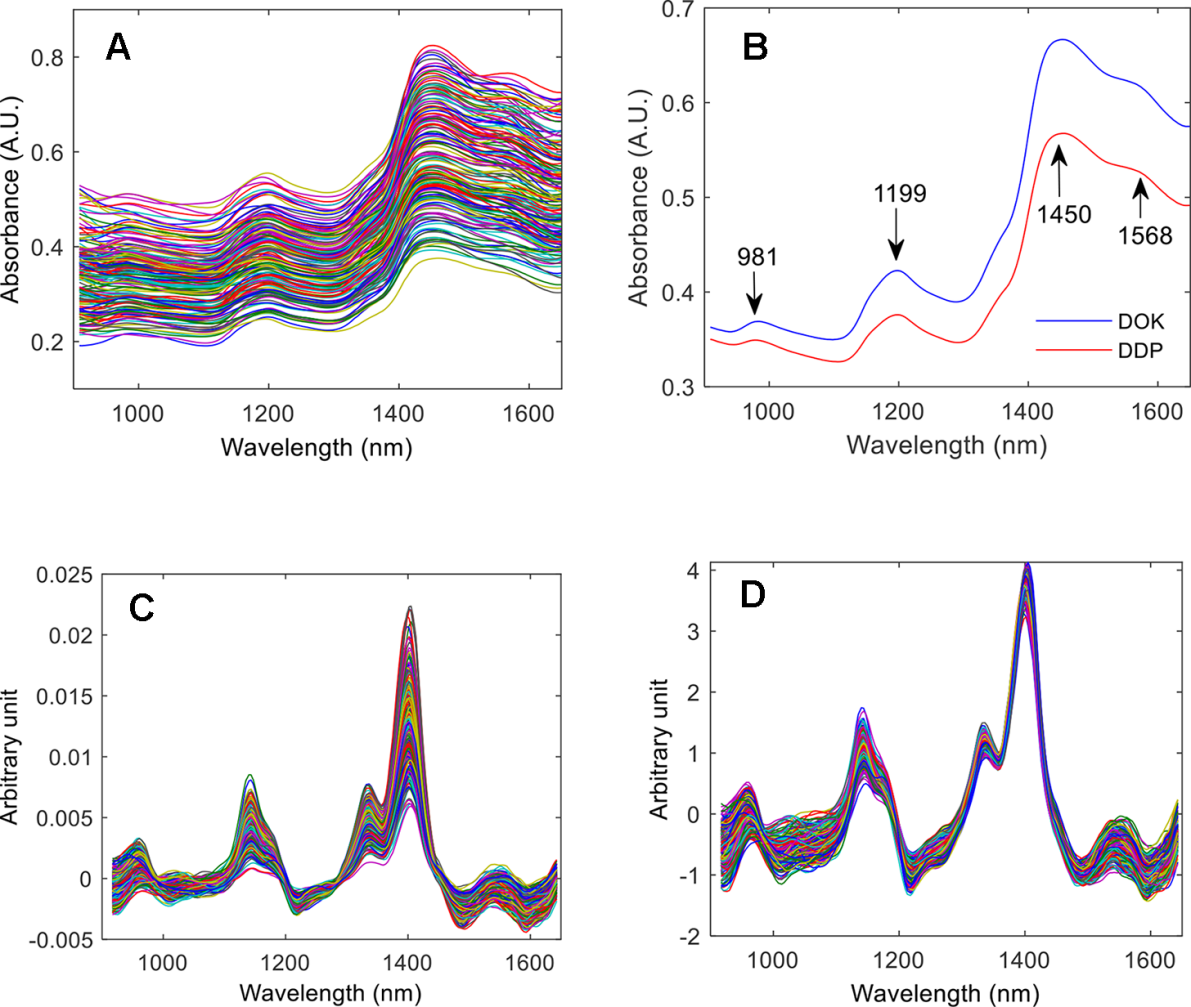
The raw NIR spectra of the fengdou calibration set **(A)**, the mean spectra of the DOK and DDP calibration samples **(B)**, the NIR spectra after pretreatment by the 1st derivative **(C)**, and the NIR spectra pretreated by the 1st derivative and subsequent SNV **(D)**.

In **Figure 4** the raw NIR spectra of the fengdou calibration set, the mean spectra of all DOK and DDP calibration samples, and the spectra of **Figure 4A** after the different pretreatment steps are shown. As can be seen from the pretreated NIR spectra in **Figures 4C, D**, the 1st derivative eliminates most of the baseline shift, whereas the SNV is applied for the scatter correction. The bands at 981 nm, 1,199 nm, and 1,450 nm can be assigned to the 2nd overtones of the N‒H, C‒H and O‒H stretching vibrations, respectively, while the band at 1,568 nm is the 1st overtone of the N‒H stretching vibration.

**Figure 5:**
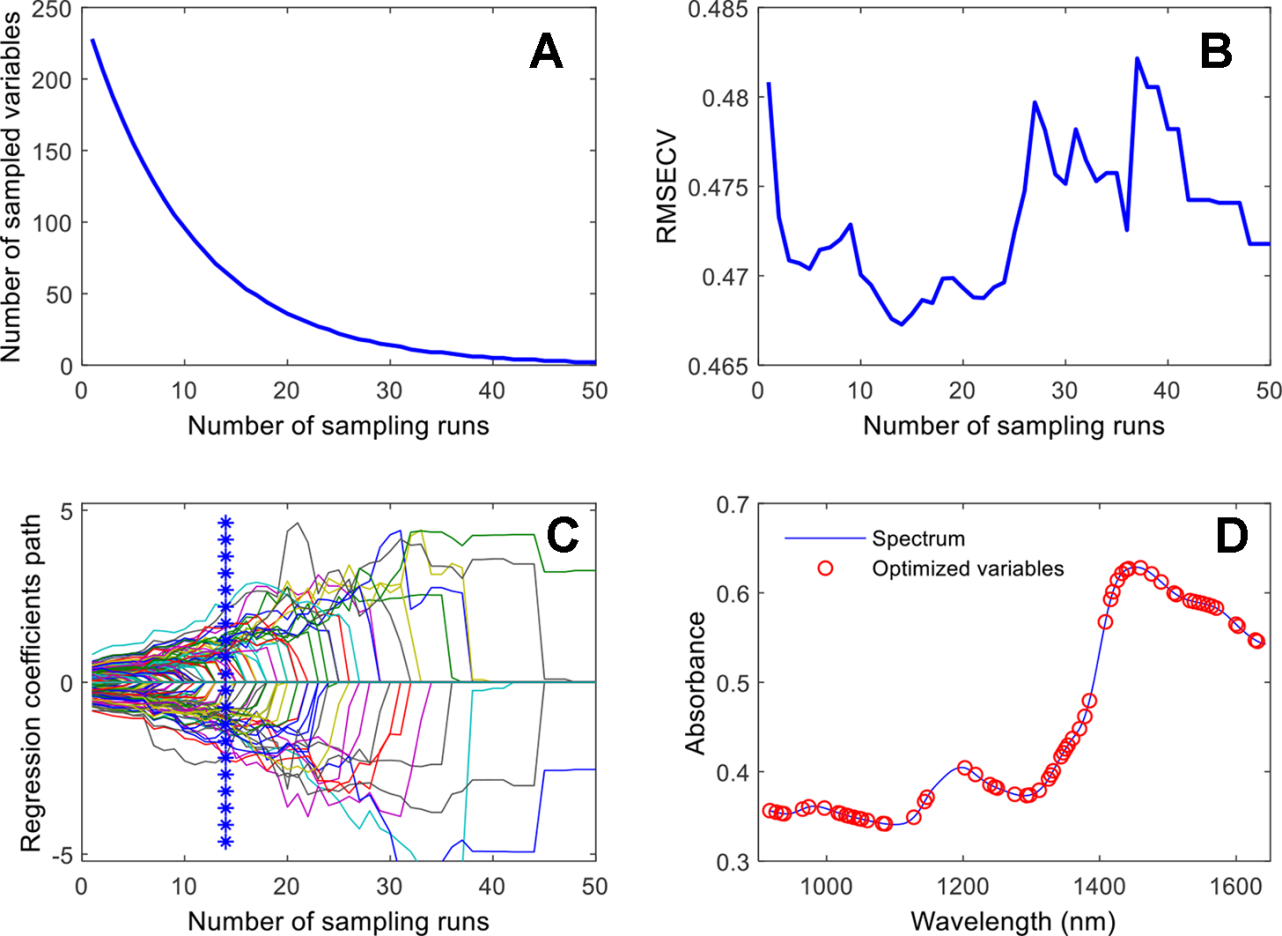
Wavelength-variable screening by CARS: **(A)** number of sampled variables versus number of sampling runs; **(B)** RMSECV versus number of sampling runs; **(C)** regression coefficients path versus number of sampling runs; **(D)** wavelength variables selected by CARS.

The diagrams of the wavelength optimization variable screening are shown in **Figures 5A–C**. As the number of sampling operations increase, the number of selected wavelength variables decreases first gradually, and then quickly. It embodies the algorithm’s ability of an initial rough selection followed by a fine-tuning (**Figure 5A**). The gradual zone for the RMSECV screening process indicates that wavelength variables irrelevant to the type of fengdou were removed, and the growth zone indicates that the essential variables relating to the type of fengdou were excluded. Finally, the trend of the regression coefficient of each wavelength variable in the screening process was achieved. The position of "*" in the figure corresponds to the minimum value of the RMSECV (**Figure 5C**). The 65 selected variables, finally selected for the calibration procedure, are shown in **Figure 5D**.

##### Identification of DOK

After spectral pretreatment by 1st derivative, SNV and mean centering, the CARS wavelength optimization algorithm was used to filter out the wavelengths with high information, and then the optimized wavelength variables were used to develop a classification model with the PLS-DA method.

**Figure 6:**
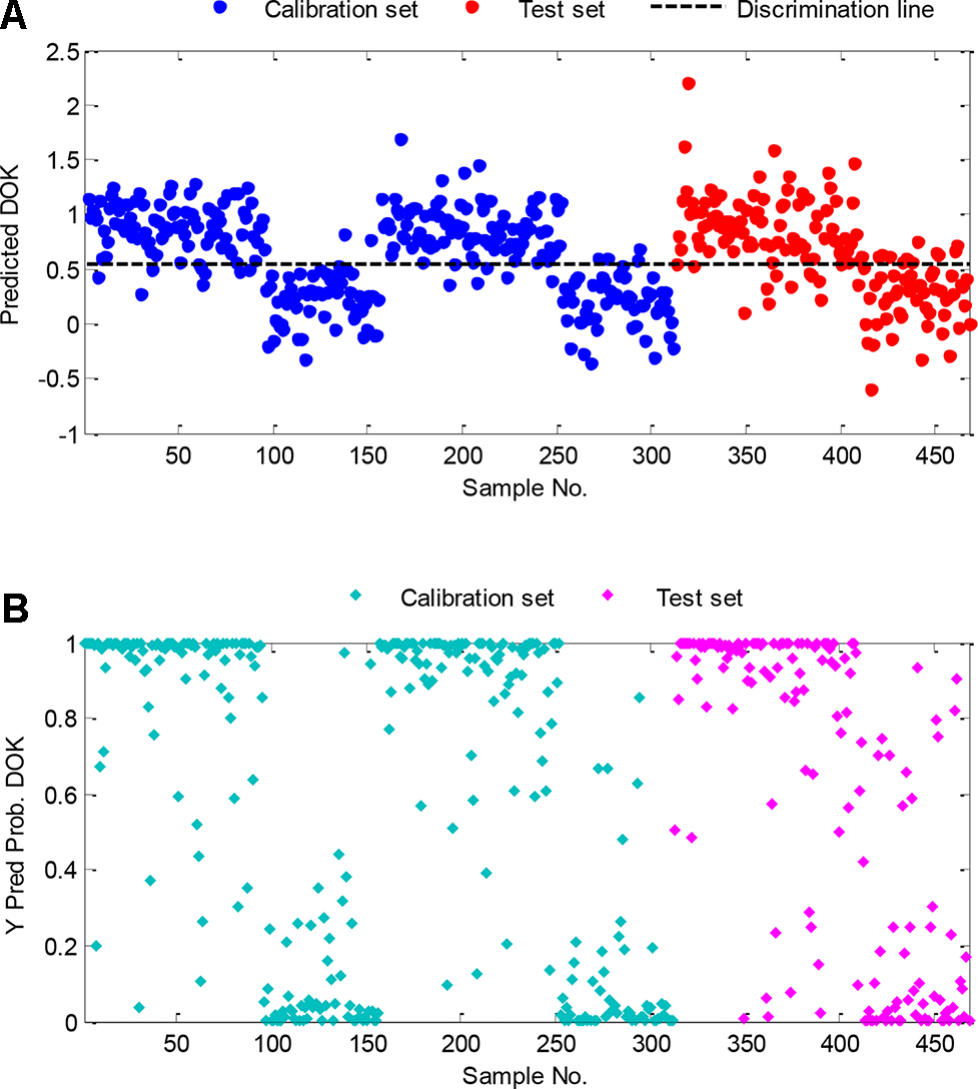
Classification results of the PLS-DA method: **(A)** DOK classification results of the calibration and test set, **(B)** classification probability of DOK of the calibration samples.

The results showed that for the calibration, cross-validation and prediction sets the accuracy is 93.9%, 89.6%, and 84.1%, respectively (**Figure 6A**). As shown by the blue dots (calibration set) and the red dots (test set) in this graph, the samples clearly cluster in two categories and can be readily discriminated. Furthermore, the probabilities of being identified as DOK were calculated and summarized in **Figure 6B**. For the majority of samples, the probability was 1 or 0, which means that these samples were either DOK or DDP. Probability values >0.5 or <0.5 refer to DOK or DDP, respectively.

Sensitivity and specificity are statistical measures of the performance of a binary classification test and are very important for qualitative analysis. Sensitivity (also called the true positive rate) measures the proportion of actual positives that are correctly identified as such. Specificity (also called the true negative rate), on the other hand, measures the proportion of actual negatives that are correctly identified. In this study, for the calibration set, cross-validation set, and test set, the sensitivities are 0.927, 0.875, 0.896, and the corresponding specificities are 0.950, 0.917, 0.783, respectively.

**Figure 7:**
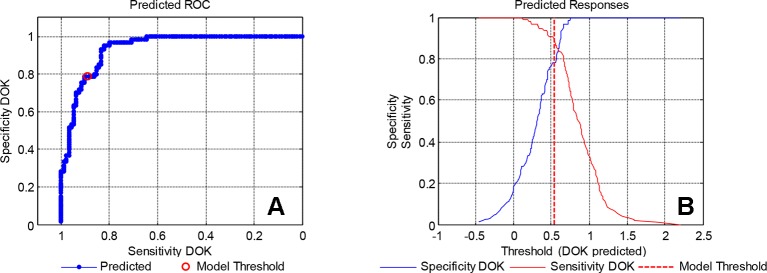
Specificity and sensitivity of the calibration model: **(A)** predicted ROC, **(B)** predicted responses.

The sensitivity and specificity derived from the PLS-DA model for the test set samples are represented in **Figure 7**. In **Figure 7B**, the threshold value used to classify the DOK is drawn as a dashed line. With the increase of the threshold value, the specificity increases, i.e., the number of false-positives DECREASES. Likewise, a sensitivity decrease represents the INCREASE of the false-negatives. With the receiver operator characteristic curve (ROC) graph in **Figure 7A** similar information is provided in a different format. The presented results, clearly demonstrate that handheld spectroscopy, combined with CARS-PLS-DA data evaluation, can be utilized for the rapid discrimination of fengdous produced from DOK or DDP.

### Quantitative Analysis of Mulberry Fruits

The mulberry fruits have a bumpy surface, and because of the fruits’ tightly-packed and seed-bearing ovaries, they have a superficial resemblance to blackberries (Huang et al., 2011). The mulberry fruits are eaten, mostly unprocessed, in their fresh state. As traditional Chinese medicine, the fresh mulberry fruit is used in the treatment of sore throats, fever, hypertension, and anemia (Kamiloglu et al., 2013); they are also used widely in the production of jams, pies, tarts, marmalades, juices, wines, and liquors, natural dyes and in the pharmaceutical, food and cosmetic industry (Huang et al., 2011; Khalifa et al., 2018).

Mulberry fruits contain high nutrient and bioactive contents, including soluble solids content (SSC), polyphenols, flavonoids, ascorbic acid, fatty acids, minerals, and anthocyanin (Lou et al., 2012). The SSC and dry matter content (DMC) are closely related to senses and nutrition. polyphenols and flavonoids (contained in polyphenols) have many pharmacological effects. polyphenols are naturally secreting, and biologically active substances and a wide range of polyphenols are provided by mulberry fruits such as flavanols, phenolic acids, derivatives, and anthocyanins. Polyphenols show activities of antioxidant, detoxification, induction of apoptosis, antiangiogenic and antiproliferation, and so on (Khalifa et al., 2018). Polyphenols in mulberry fruits and their corresponding functionalities vary considerably according to the genetic diversity, climatic, agricultural practices, processing conditions, and stability during storage (Khalifa et al., 2018). Flavonoids are found mostly in glycosylated form, and they have complex flavonol glycosides profiles including 13 quercetin derivatives, five kaempferol derivatives, and O-methylated flavonol-analogs, such as rhamnetin and isorhamnetin. Levels of quercetin glycoside are reported to increase as the fruit ripens from white to black stages (Sánchez-Salcedo et al., 2015). The flavonoids variation in different breeds of mulberries is significant (Sánchez-Salcedo et al., 2015).

Fruit quality has traditionally been determined by visual inspection of the external appearance and its internal content determined by destructive methods, which require operators with the expertise to perform the analysis in a professional laboratory. However, this is impractical for routine analysis by ordinary people. In recent times, consumers have grown conscious of the health benefits of the ingredients of this fruit and a new approach to determine their concentrations is required. In this context it has been reported, that NIR spectroscopy can be used to nondestructively analyze the internal contents, including the SSC, DMC, and total polyphenol content (TPC) of apples (Pissard et al., 2012). Furthermore, Chen et al. employed FT-NIR spectroscopy to determine the TPC in green tea (Chen et al., 2008). In view of this prior knowledge, the demand for a new analytical procedure of mulberry fruits, that will require little to no training originated. In the present work, this issue is addressed by applying the hand-held NIR spectrometer MicroNIR 1700 for a feasibility study of the fast determination of SSC, DMC, polyphenols, and flavonoids in fresh mulberry fruits.

#### Materials and Methods

##### Samples

The mulberry varieties applied in this work are Zhongmu 1, 8632, Mengchang 4, and Dashi. A total of 434 mulberry fruits (6–9 maturity) were collected from the conservation of mulberry germplasm resources of the Institute of Sericulture, Chinese Academy of Agricultural Sciences (Zhenjiang, Jiangsu, China).

##### Measurement of NIR Spectra

**Figure 8:**
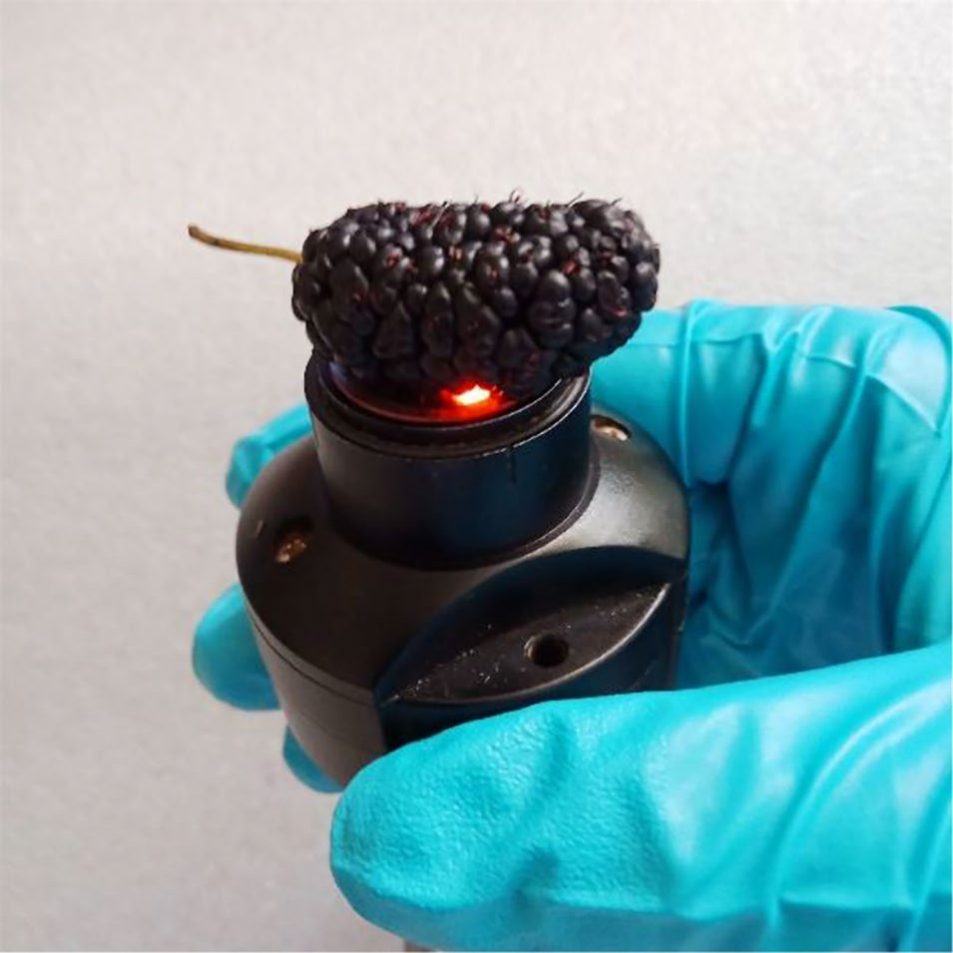
Presentation of the mulberry fruit for NIR spectra measurement with the MicroNIR 1700.

As shown in **Figure 8**, NIR diffuse reflection spectra of mulberry fruits were collected with the MicroNIR 1700 spectrometer by accumulating 50 scans with an integration time of 15 ms, and 125 wavelength variables in the range from 908 to 1,676 nm. Triplicate measurements were made at different spots, and the average of the three spectra was used as the final spectrum of the sample for further processing. The measurements were performed at an environmental temperature of 25 °C and a humidity of about 40%.

##### Reference Analysis

###### Determination of Soluble Solids Content

After collection of the NIR spectra, the SSC was determined immediately by a refractometer. First, the equipment was calibrated to zero with distilled water, then the detection surface was dried, and then a few drops of mulberry fruit juice were applied to the detection surface. The juice drops were spread on the prism surface by gently closing the cover of the refractometer, and the corresponding refractive index value was taken.

###### Determination of Dry Matter Content

The DMC was obtained by measuring the weight percentage of the dried fruit against the corresponding value of the fresh fruit. The weight of the fresh mulberry fruit was measured as m^1^, and then the fruit was dried at 65 °C for 24 h and finally dried to constant weight m^2^ at 105 °C. The DMC was calculated as DMC (%) = (m^2^/m^1^) × 100 (%).

###### Determination of Total Polyphenol Content

The TPC of mulberry fruit was determined by the Folin–Ciocalteau method (Yu and Dahlgren, 2000).

###### Determination of Total Flavonoid Content

The content of total flavonoids content (TFC) in the investigated mulberry fruits was measured by colorimetry (Marinova et al., 2005).

##### Evaluation of Spectra

###### Spectral Pretreatment

The standard normal variate (SNV) transformation and the 1st derivative based on a Savitzky Golay smoothing procedure with a five data point window and a 2nd order polynomial were applied.

###### Wavelength Optimization

In this work, two kinds of wavelength selection methods have been applied: genetic algorithm (GA) and CARS. GA is an adaptive search procedure based on the mechanism of genetics and natural selection (Shao et al., 2004; Yan et al., 2011). At first, the GA algorithm randomly generates a population (each individual in the population represents a way of solving the problem) that is composed of a binary string (called chromosome). The bit value “1” represents a selected variable whereas “0” is a variable that is not selected. The fitness of an individual (its ability to adapt to the environment) is calculated; high-quality individuals are retained, low-quality individuals are out. New individuals are generated through inheritance and evolved through natural selection. In this way, eventually, the solution of the problem is achieved. In the present work, the parameters chromosomes 30, mutation 1% and cross-over 50% were adopted in the GA to optimize the variables. The principle of the CARS technique has been described for the previous application example and will not be repeated here.

###### PLS Calibration

PLS calibration was developed using the PLS toolbox (version 6.21, Eigenvector Inc., Manson, WA, USA), and internal cross-validation (CV) was used to select the optimum number of factors. CV estimated the prediction error by splitting all samples into 20 segments, and one segment was reserved for validation, and the remaining (Næs et al., 2002) segments were used for calibration. This process was repeated until all segments were used for validation once.

###### Calibrations and Validation Statistics

Calibration and validation statistics included the RMSEof calibration set (RMSEC), RMSECV and RMSE of prediction set (RMSEP) and R-squares (Fan et al., 2016). The RMSEC, RMSECV, and RMSEP were used to evaluate the feasibility of the model and its predictive ability. The lower the RMSEP and the closer its value to the RMSEC, the stronger is the prediction ability, and the greater is the robustness of the model. The residual predictive deviation (RPD) defined by the Std Dev/RMSEC of the calibration set was also included to estimate how well the calibration model can predict the compositional data. Generally, an RPD value greater than three can be considered as very good for prediction purposes (Fearn, 2002).

##### Validation With Unknown Samples

Unknown mulberry fruit samples were collected as a test set to validate the prediction capability of the calibration models developed for SSC, DMC, TPC, and TFC.

### Results and Discussion

*Reference Values*. The reference values of SSC, DMC, TPC, and TFC in mulberry fruits were determined after the spectra were recorded. As shown in **Table 1**, the mean of SSC, DMC, TPC, and TFC were 10.21 Brix, 11.92%, 3.06 mg/g, and 2.26 mg/g, respectively, and the corresponding standard deviation values were 3.16 Brix, 2.26%, 1.25 mg/g and 0.84 mg/g, respectively. The coefficients of variation (C.V.) were 30.96%, 18.94%, 40.95%, and 37.32%, respectively, which suggested that the parameters vary strongly, especially for the TPC and TFC. It is indicative that the collected samples are representative, and the calibration model will show good performance for the determination of unknown samples.

**Table 1:** Statistical analysis of the reference results of the 4 parameters of mulberry fruits.

Statistical Parameters	SSC (Brix)			DMC(g/g, %)			TPC (mg/g)			TFC(mg/g)		
	Total	Cal.*	Test *	Total	Cal.	Test	Total	Cal.	Test	Total	Cal.	Test
Number	113	76	37	94	63	31	91	61	30	81	54	27
Mean	10.16	10.21	10.07	11.94	11.92	11.87	3.07	3.06	3.09	2.34	2.26	2.51
Max	17.39	17.39	16.37	16.54	16.54	16.43	6.67	6.67	6.48	4.01	4.01	3.98
Min	3.80	3.80	4.00	7.87	7.17	7.87	0.97	1.22	0.97	1.07	1.07	1.09
Range	13.59	13.59	12.37	8.67	9.37	8.55	5.70	5.45	5.51	2.94	2.94	2.90
Std.	3.10	3.16	3.02	2.29	2.26	2.35	1.23	1.25	1.19	0.83	0.84	0.78
C.V.	30.51	30.96	29.94	19.20	18.94	19.81	39.93	40.95	38.50	35.28	37.38	30.97

*Cal and Test stand for calibration and test set, respectively.

### NIR Spectra

**Figure 9:**
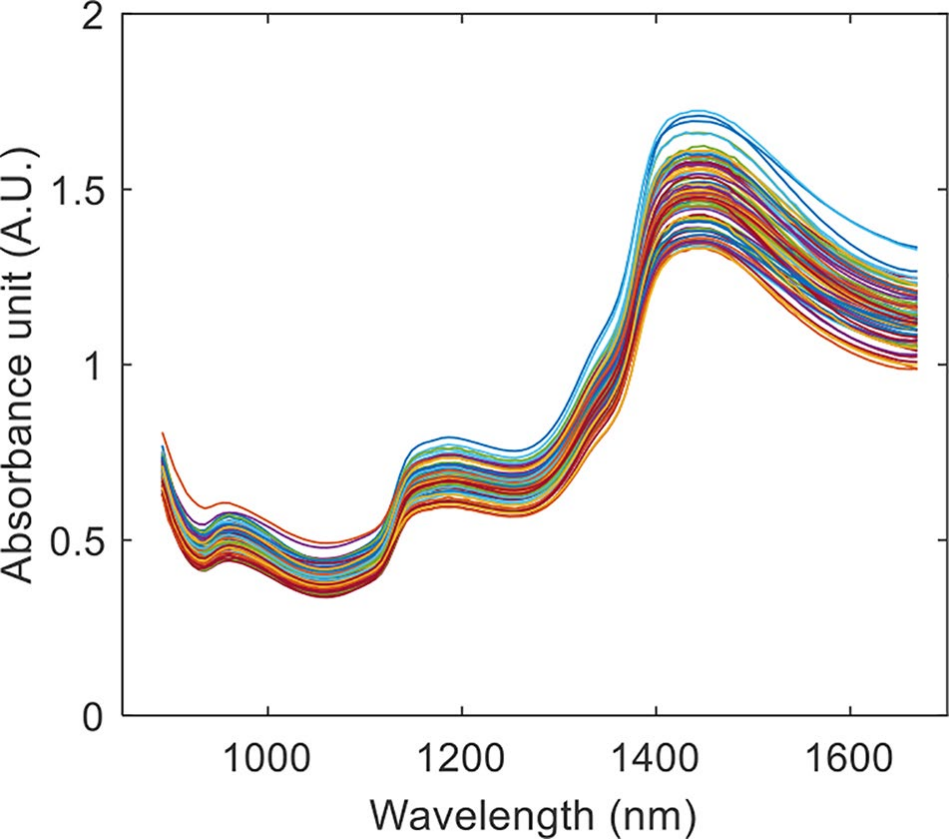
The raw NIR spectra of the mulberry fruit calibration set.

The raw NIR spectra of the calibration set are shown in **Figure 9**. The absorption bands at 990 nm and 1,450 nm are related to the 2nd and 1st overtones of the ν(OH) stretching vibration, respectively. The absorption bands from 1,110 nm to 1255 nm belong to the 2nd overtones of ν(CH) stretchng vibrations.

### Spectral Pretreatment

**Figure 10:**
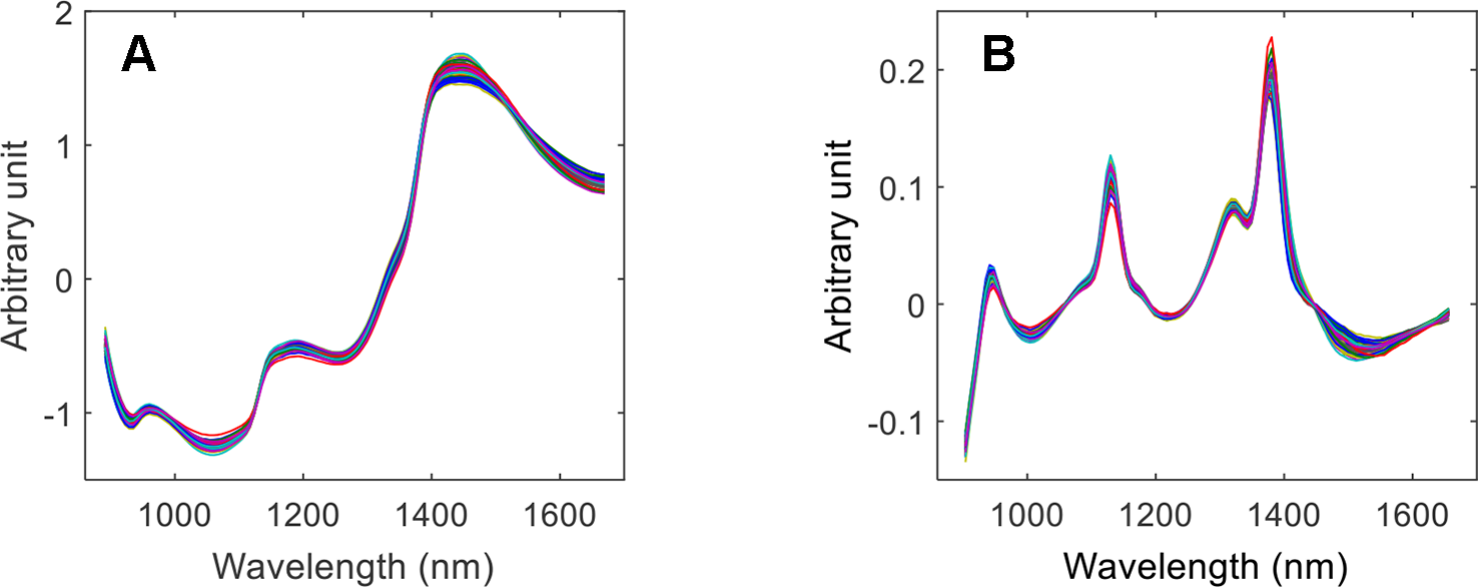
Calibration spectra pretreated by SNV **(A)**, and by SNV + 1st derivative **(B)**.

Different methods were used to pretreat the spectral data. The spectra pretreated by SNV only, and a combination of SNV + 1st derivative are shown in **Figures 10A, B**, respectively, and specifically in the second pretreatment, an accentuation of spectral features can be observed.

The results in **Table 2** show that the pretreated spectra can significantly affect the prediction accuracy of the model. Because the SNV method corrects for scattering effects caused by sample roughness and particle heterogeneity (Yan and Siesler, 2018b) the prediction accuracy of the SSC and DMC calibration models is improved. For the TPC and TFC, the SNV followed by the 1st derivative yielded the best calibration performance. Obviously, besides the scatter correction effect of the SNV, the first derivative contributes spectral features that are beneficial for the calibration of low-content and complex components (such as the polyphenols and flavonoids).

**Table 2:** The influence of spectra pretreatment methods on the calibration performance (the best calibration results are reproduced in bold numbers).

Parameters	Pretreatment Methods	Factors	R_c_^2^	RMSEC	R_cv_^2^	RMSECV	R_p_^2^	RMSEP
SSC	None	9	0.9142	0.9198	0.872	1.1931	0.9001	0.9667
	**SNV**	**7**	**0.9129**	**0.9266**	**0.8883**	**1.0962**	**0.8891**	**1.0412**
	SNV + 1st	7	0.918	0.8989	0.8867	1.1041	0.8974	1.0168
	1st	7	0.9112	0.9358	0.8751	1.2263	0.8781	1.076
	1st + SNV	6	0.9113	0.9352	0.8856	1.1038	0.8879	1.059
DSC	None	7	0.9016	0.7031	0.8409	0.9695	0.8613	1.0215
	**SNV**	**7**	**0.9148**	**0.654**	**0.8683**	**0.8806**	**0.9164**	**0.7328**
	SNV + 1st	7	0.9324	0.5825	0.8894	0.8163	0.8962	0.8901
	1st	7	0.9119	0.665	0.8621	0.8918	0.8919	0.9753
	1st + SNV	5	0.8703	0.8072	0.8277	0.9656	0.8647	0.9776
TPC	None	7	0.8176	0.5307	0.7312	0.688	0.8288	0.5764
	SNV	7	0.8675	0.4524	0.8057	0.5917	0.8485	0.5184
	**SNV + 1st**	**6**	**0.8829**	**0.4253**	**0.8343**	**0.5364**	**0.8385**	**0.537**
	1st	6	0.865	0.4567	0.8165	0.5703	0.8422	0.5568
	1st + SNV	5	0.8301	0.5123	0.7558	0.65	0.8395	0.5722
								
TFC	None	7	0.7733	0.3977	0.6632	0.52	0.5665	0.5717
	SNV	7	0.8146	0.3596	0.7124	0.48	0.7027	0.4662
	**SNV + 1st**	**6**	**0.8249**	**0.3494**	**0.7203**	**0.4737**	**0.7364**	**0.4023**
	1st	6	0.8088	0.3652	0.7208	0.4751	0.5864	0.5625
	1st + SNV	6	0.8292	0.3451	0.7431	0.4567	0.7116	0.417

### Wavelength Selection

**Figure 11:**
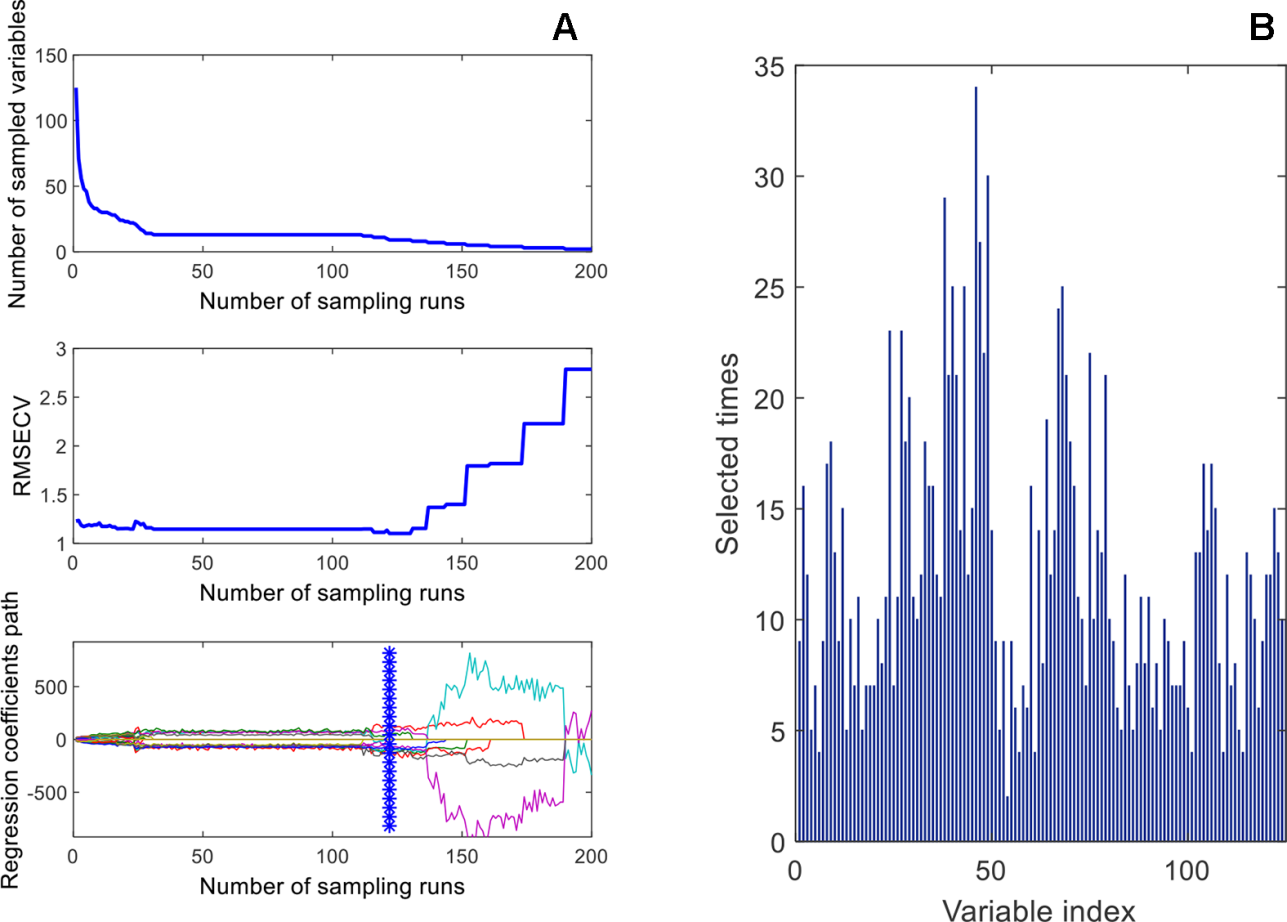
Wavelength-variable screening by CARS **(A)**, and GA **(B)**.

**Figure 12:**
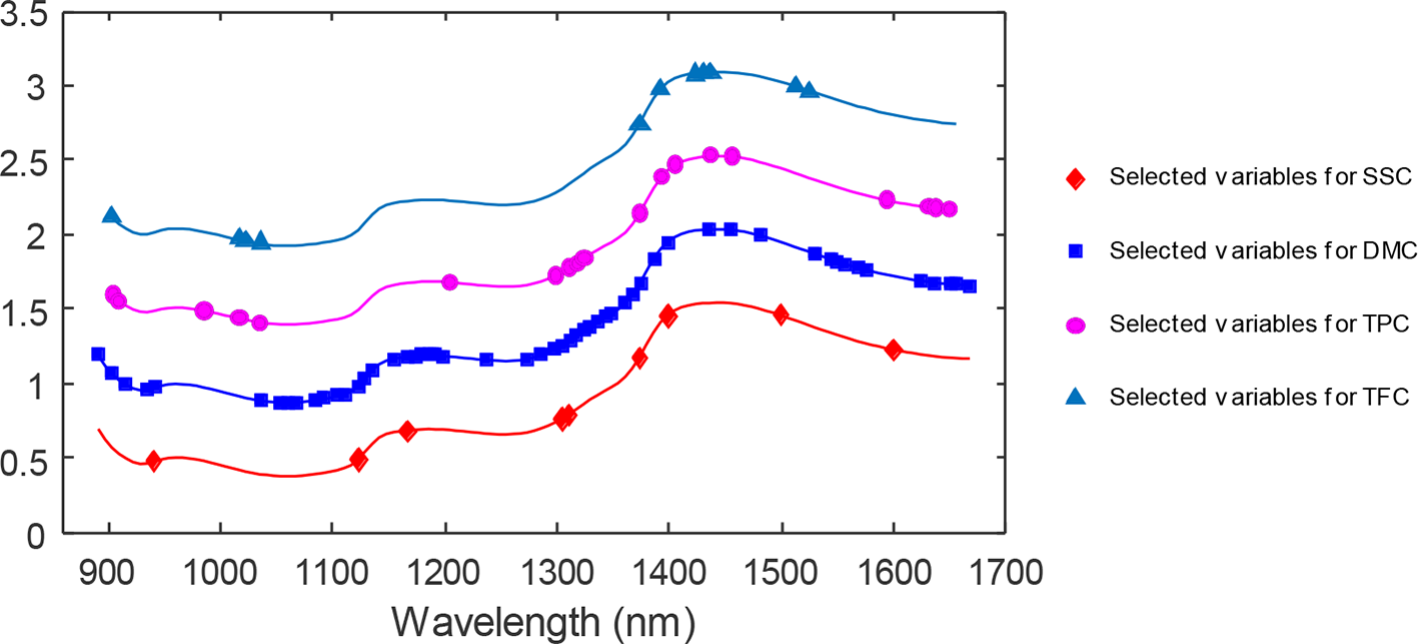
The wavelength variables selected by CARS (♦, •, ▴) and GA (▪) for the four parameters under investigation.

**Figure 11A** shows a diagram of the NIR wavelength selection screening for the SSC content that is similar to the previous application example. By the CARS selection, the most sensitive wavelength variables were obtained (see **Table 3**). For SSC, TPC, and TFC, the performance of CARS was better than that of GA. As shown in **Figure 11B** for DMC, 54 variables were selected in 200 runs of the genetic algorithms and subsequently used for the development of a PLS model. The different variables selected by these two methods for the four components are shown in **Figure 12**. It is of interest that the variables at about 900 nm, 1,110 nm and in the 1,380–1,440 nm range, selected for TFC are also selected for TPC; the reason maybe that the flavonoids belong to the class of polyphenols and these variables are important for both, TPC and TFC.

**Table 3:** Comparison of the impact of the two wavelength selection methods CARS and GA on the calibration performance of the four quality parameters of mulberry fruits (the bold numbers highlight the best calibration results).

Methods	Parameters	Variables	Factors	R_c_^2^	RMSEC	R_cv_^2^	RMSECV	RPD	R_p_^2^	RMSEP
Cars	SSC	**9**	**5**	**0.9179**	**0.8998**	**0.8979**	**1.0462**	**3.51**	**0.9313**	**0.8843**
	DMC	10	4	0.9036	0.6957	0.8842	0.7841	3.25	0.9194	0.7961
	TPC	**19**	**5**	**0.8989**	**0.3952**	**0.8643**	**0.4818**	**3.16**	**0.8651**	**0.4884**
	TFC	**11**	**5**	**0.8154**	**0.3588**	**0.7711**	**0.412**	**2.34**	**0.7177**	**0.4061**
GA	SSC	14	7	0.9287	0.8382	0.9046	1.0108	3.77	0.9043	1.0146
	DMC	**54**	**7**	**0.9295**	**0.5950**	**0.8977**	**0.7608**	**3.80**	**0.9071**	**0.7758**
	TPC	75	6	0.8942	0.4042	0.8585	0.4916	3.09	0.8642	0.4876
	TFC	27	6	0.7914	0.3815	0.7299	0.4536	2.20	0.7153	0.4097

### Analysis of the Calibration Statistics

**Figure 13:**
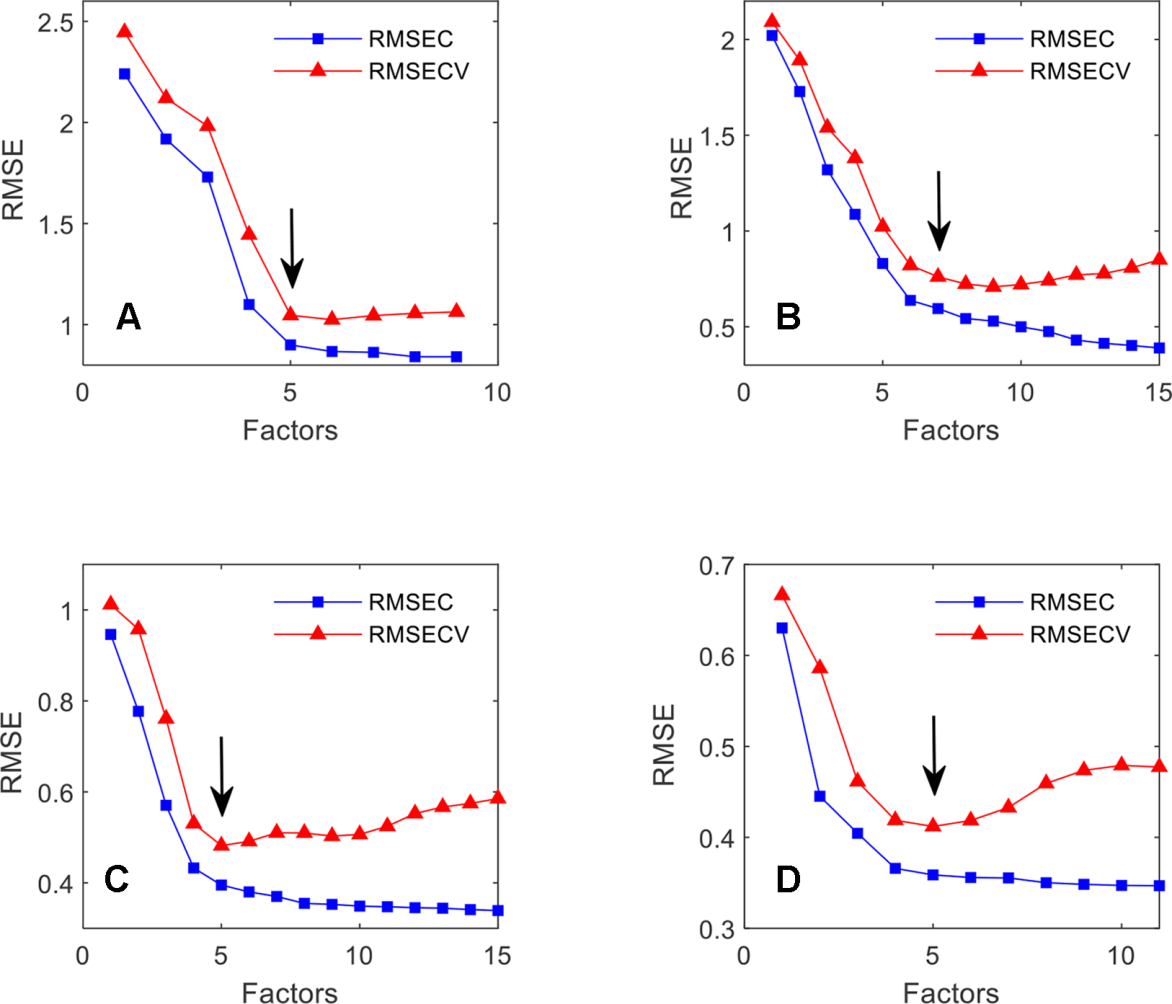
The effect of the number of factors on the RMSEC and RMSECV for SSC **(A)**, DMC **(B)**, TPC **(C)** and TFC **(D)**.

The number of optimal factors chosen for a calibration model has a significant impact on its prediction ability. When the number of factors is too low, the model does not entirely reflect the characteristics of the substance, which leads to lower prediction accuracy. Too many factors lead to over-fitting and yield an—apparently—high prediction accuracy. However, when the model is applied to unknown samples, the prediction effect is weak because the model is not robust. Cross-validation was applied to the calibration models with the smallest optimal number of factors. For SSC, DSC, TPC, and TFC, the optimal number of factors are 5,7,5 and 5, respectively. In **Figure 13** the graphs of the RMSEC and RMSECV versus the number of factors are shown for the SSC, DMC, TPC, and TFC. The errors mark the final choice of the optimum number of factors for the individual parameter.

The calibration parameters for the different components are summarized in **Table 3**. Although only nine wavelength variables were selected for SSC, the calibration performance is the highest. The R_c_^2^ and R_cv_^2^ are 0.9179 and 0.8979, and the corresponding RMSEC and RMSECV are 0.8998 Brix and 1.0462 Brix, respectively. The high R^2^ values and the low RMSEs are characteristic of a good prediction capability. Furthermore, the R^2^ and RMSE values for the calibration and cross-validation are similar, which indicates that the calibration model is robust. For DMC, the best calibration is built with the 54 wavelength variables selected by GA. The R^2^ values for the calibration and cross-validation are 0.9295 and 0.8977, respectively, and the corresponding RMSEC and RMSECV are 0.5950% and 0.7608%, also suggesting a good calibration performance. However, the robustness is not as good as that of the SSC calibration, because of the larger difference between the statistical parameters of the calibration and the cross-validation. For TPC, 19 wavelength variables were selected for the calibration, and the R^2^ values are not as high as that of the DMC calibration. Therefore, the calibration yields results of lower accuracy than the DMC calibration, and furthermore, its robustness is also lower. Finally, the performance of the TFC calibration with 11 wavelength variables is also not as high as that of the TPC component. The R_c_^2^ and R_cv_^2^ are 0.8154 and 0.7711, respectively, with the consequence of lower calibration accuracy. The RPD values are also included to estimate how well the calibration model can predict the compositional data (Williams and Sobering, 1993; Fearn, 2002). The RPDs for SSC, DMC, TPC, and TFC are 3.77, 3.80, 3.16 and 2.34, respectively, which furnish evidence that SSC, DMC, and TPC can be accurately predicted in the investigated concentration range, whereas, at best, a medium quality calibration has been achieved for TFC.

**Figure 14:**
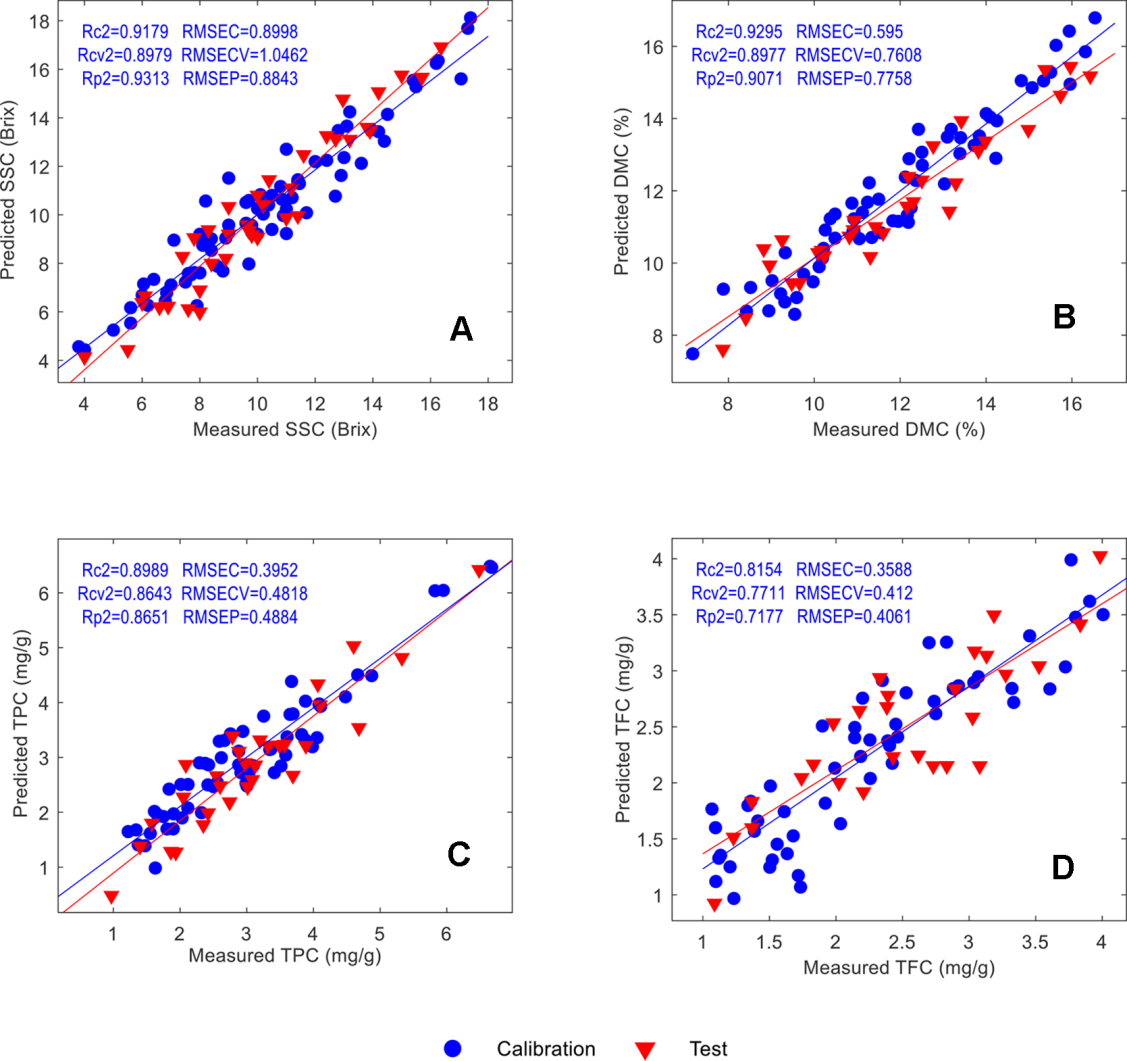
Scatter plots of the measured and predicted parameter values of the calibration and test samples: SSC **(A)**, DMC **(B)**, TPC **(C)** and TFC **(D)**.

The scatter plots of the measured versus the predicted parameters are shown in **Figure 14**. In agreement with the previously discussed calibration statistics results, the scatter distances from the regression lines also reflect that proper calibrations have been developed for SSC, DMC and TPC whereas for TFC a comparatively lower calibration performance has been achieved.

### Validation With Test Samples

In order to test the performance of the calibrations, a series of test samples (defined as “unknowns” despite available reference values) were used to validate the prediction accuracy. Their calibration statistics results have been summarized in **Table 3**. The R_p_^2^ for SSC, DMC, TPC and TFC are 0.9313, 0.9071, 0.8651 and 0.9071, respectively, and the corresponding RMSEPs are 0.8843 Brix, 0.7758 %, 0.4884 mg/g and 0.4061 mg/g, respectively. The similar accuracy for the calibration set and cross-validation set suggests that the calibrations are robust. A detailed comparison of prediction and reference results is provided in **Table 4**. In general, for the SSC and DMC, the absolute and relative errors are small, which meets the application requirements. Large relative errors were obtained for the TPC and TFC, but because the absolute errors are small, the calibrations are suitable for screening purposes of consumers, who use a handheld NIR spectrometer to detect whether the mulberry fruits contain a high content of TPC or TFC that is beneficial for the human body.

**Table 4:** Prediction results for the “unknown” test samples.

Parameters	No.	Measured	Predicted	Absolute Error	Relative Error(%)	No.	Measured	Predicted	Absolute Error	Relative Error(%)
SSC (Brix)	S1	4.00	4.14	0.14	3.57	S20	10.00	10.80	0.80	8.00
	S2	5.50	4.44	−1.06	−19.30	S21	10.20	10.41	0.21	2.06
	S3	6.00	6.37	0.37	6.24	S22	10.40	11.44	1.04	10.00
	S4	6.10	6.65	0.55	9.02	S23	10.00	9.07	−0.93	−9.30
	S5	6.60	6.20	−0.40	−6.09	S24	13.90	13.46	−0.44	−3.17
	S6	6.90	6.23	−0.67	−9.77	S25	11.00	9.87	−1.13	−10.27
	S7	7.40	8.28	0.88	11.89	S26	11.20	11.10	−0.10	−0.89
	S8	7.60	6.12	−1.48	−19.48	S27	11.40	9.96	−1.44	−12.63
	S9	7.80	9.05	1.25	16.05	S28	11.60	12.47	0.87	7.50
	S10	8.00	6.90	−1.10	−13.78	S29	12.40	13.26	0.86	6.94
	S11	8.00	5.99	−2.01	−25.17	S30	12.70	13.14	0.44	3.46
	S12	8.30	9.38	1.08	12.99	S31	12.90	14.76	1.86	14.42
	S13	8.40	8.00	−0.40	−4.81	S32	13.20	13.10	−0.10	−0.76
	S14	8.90	8.20	−0.70	−7.85	S33	13.80	13.60	−0.20	−1.45
	S15	9.00	10.33	1.33	14.82	S34	14.20	15.07	0.87	6.13
	S16	9.00	9.20	0.20	2.21	S35	15.00	15.75	0.75	5.00
	S17	9.60	9.58	−0.07	−0.72	S36	15.70	15.66	-0.04	−0.25
	S18	9.70	9.34	−0.02	−0.20	S37	16.40	16.92	0.52	3.17
	S19	9.80	9.17	−0.63	−6.41					
DMC (%)	D1	7.87	7.61	−0.27	−3.37	D17	12.16	11.57	−0.59	−4.83
	D2	8.40	8.48	0.08	0.90	D18	12.19	12.39	0.20	1.63
	D3	8.82	10.39	1.57	17.79	D19	12.31	11.69	−0.61	−4.98
	D4	8.96	9.94	0.98	10.94	D20	12.51	12.29	−0.21	−1.72
	D5	9.25	10.64	1.39	15.00	D21	12.77	13.24	0.47	3.71
	D6	9.47	9.45	−0.03	−0.29	D22	13.14	11.43	−1.71	−13.04
	D7	9.66	9.45	−0.21	−2.14	D23	13.29	12.21	−1.08	-8.13
	D8	10.09	10.28	0.20	1.96	D24	13.42	13.94	0.51	3.83
	D9	10.17	10.34	0.18	1.74	D25	13.82	13.11	−0.71	−5.15
	D10	10.23	10.20	−0.03	−0.33	D26	13.99	13.37	−0.62	−4.40
	D11	10.82	10.74	−0.08	−0.76	D27	14.99	13.69	−1.29	−8.63
	D12	10.91	10.92	0.01	0.05	D28	15.38	15.35	−0.02	−0.15
	D13	10.93	11.18	0.25	2.28	D29	15.74	14.64	−1.10	−6.96
	D14	11.31	10.17	−1.13	−10.02	D30	15.96	15.44	−0.52	−3.28
	D15	11.42	11.00	−0.42	−3.70	D31	16.43	15.18	−1.25	−7.61
	D16	11.60	10.83	−0.77	−6.63					
TPC (mg/g)	P1	2.05	2.27	0.23	11.01	P16	3.01	2.46	−0.55	−18.29
	P2	1.58	1.80	0.22	14.04	P17	3.08	2.59	−0.49	−15.89
	P3	1.40	1.38	−0.02	−1.38	P18	3.13	2.86	−0.27	−8.61
	P4	1.86	1.28	−0.58	−31.05	P19	3.32	3.22	−0.10	−2.92
	P5	1.94	1.27	−0.66	−34.30	P20	3.49	3.22	−0.27	−7.63
	P6	2.09	2.87	0.78	37.45	P21	3.57	3.24	−0.33	−9.26
	P7	3.20	3.32	0.12	3.71	P22	3.69	2.67	−1.02	−27.65
	P8	2.35	1.77	−0.58	−24.63	P23	3.89	3.22	−0.67	−17.29
	P9	2.42	1.99	−0.43	−17.85	P24	4.07	4.34	0.27	6.66
	P10	2.55	2.66	0.11	4.41	P25	4.10	3.96	−0.14	−3.44
	P11	2.60	2.48	−0.12	−4.67	P26	4.60	5.03	0.43	9.38
	P12	2.74	2.19	−0.56	−20.28	P27	4.68	3.54	−1.14	−24.37
	P13	2.79	3.39	0.60	21.58	P28	5.33	4.82	−0.51	−9.54
	P14	2.88	3.11	0.22	7.78	P29	6.48	6.42	−0.06	−0.97
	P15	2.97	2.90	−0.07	−2.43	P30	0.97	0.48	−0.49	−50.44
TFC (mg/g)	F1	1.74	2.05	0.30	17.46	F15	2.90	2.84	−0.06	−2.00
	F2	1.98	2.53	0.55	27.98	F16	3.04	3.17	0.13	4.39
	F3	3.13	3.14	0.01	0.17	F17	3.84	3.42	−0.42	−10.92
	F4	2.18	2.64	0.47	21.52	F18	1.37	1.83	0.46	33.80
	F5	2.21	1.92	−0.29	−13.04	F19	2.02	2.00	−0.02	−1.04
	F6	2.33	2.94	0.61	26.17	F20	2.73	2.15	−0.58	−21.23
	F7	2.39	2.78	0.39	16.30	F21	3.19	3.50	0.31	9.76
	F8	2.43	2.23	−0.20	−8.08	F22	3.28	2.97	−0.31	−9.37
	F9	2.62	2.25	−0.37	−14.23	F23	1.37	1.60	0.23	16.66
	F10	2.83	2.15	−0.68	−24.00	F24	1.09	0.92	−0.16	−15.07
	F11	3.03	2.59	−0.44	−14.58	F25	2.38	2.68	0.30	12.45
	F12	3.08	2.15	−0.93	−30.16	F26	1.83	2.17	0.33	18.30
	F13	3.53	3.04	−0.48	−13.67	F27	1.23	1.51	0.28	22.99
	F14	3.98	4.03	0.04	1.06					

## Conclusions

Generally, hand-held NIR instruments have launched vibrational spectroscopy into a new era of in-the-field and on-site analysis. In the present communication hand-held NIR spectrometers were applied for qualitative and quantitative plant analytical case studies. In the qualitative example, it was demonstrated that high-value fengdous based on DOK plants can be successfully discriminated from lower quality fengdous of DDP plants. The quantitative application example outlined in detail the assay of the nutritional parameters SSC, DMC, TPC, and TFC of mulberry fruits by hand-held NIR spectroscopy. In both cases, the analysis of the spectroscopic data was performed with chemometric evaluation routines in combination with wavelength selection methods.

Although the measurement and evaluation routines have not yet reached the convenience for public use by a non-expert user community, the integration of NIR spectrometers into mobile phones and the development of apps for specific analytical procedures in food, plant and material quality control will significantly change the every-day-life of consumers in the near future.

## Data Availability Statement

All datasets generated for this study are included in the article/supplementary material.

## Author Contributions

HY: Investigation, Data curation, Methodology. Y-CX: Investigation, Data curation. HS: Methodology, Supervision. B-XH: Funding acquisition, Investigation. G-ZZ: Funding acquisition, Investigation.

## Funding

This Work Was Supported by a Special Project for the Construction of a Modern Agricultural Technology System (Grant Number CARS-18, CARS-21), National Key Research and Development Program of China (2017YFC1700701), Anhui Provincial Science Fund for Distinguished Young Scholars (1808085J17), Jiangsu Province Natural Science Foundation (Grant Number BK20131239).

## Conflict of Interest

The authors declare that the research was conducted in the absence of any commercial or financial relationships that could be construed as a potential conflict of interest.
